# A neuronal MCT2 knockdown in the rat somatosensory cortex reduces both the NMR lactate signal and the BOLD response during whisker stimulation

**DOI:** 10.1371/journal.pone.0174990

**Published:** 2017-04-07

**Authors:** Leslie Mazuel, Jordy Blanc, Cendrine Repond, Véronique Bouchaud, Gérard Raffard, Nicole Déglon, Gilles Bonvento, Luc Pellerin, Anne-Karine Bouzier-Sore

**Affiliations:** 1 Centre de Résonance Magnétique des Systèmes Biologiques UMR 5536, CNRS—Université Bordeaux 146 rue Léo-Saignat, Bordeaux, France; 2 Département de Physiologie, 7 rue du Bugnon, CH Lausanne, Switzerland; 3 Department of Clinical Neurosciences, Laboratory of Cellular and Molecular Neurotherapies (LCMN), Lausanne University Hospital, Lausanne, Switzerland; 4 Neurosciences Research Center (CRN), LCMN, Lausanne University Hospital, Lausanne, Switzerland; 5 Commissariat à l’Energie Atomique et aux Energies Alternatives (CEA), Département de la Recherche Fondamentale (DRF), Institut d’Imagerie Biomédicale (I2BM), Molecular Imaging Center (MIRCen), CNRS UMR 9199, Université Paris-Sud, Université Paris-Saclay, Fontenay-aux-Roses, France; Albany Medical College, UNITED STATES

## Abstract

Although several *in vitro* and *ex vivo* evidence support the existence of lactate exchange between astrocytes and neurons, a direct demonstration *in vivo* is still lacking. In the present study, a lentiviral vector carrying a short hairpin RNA (shRNA) was used to downregulate the expression of the monocarboxylate transporter type 2 (MCT2) in neurons of the rat somatosensory cortex (called S1BF) by ~ 25%. After one hour of whisker stimulation, HRMAS ^1^H-NMR spectroscopy analysis of S1BF perchloric acid extracts showed that while an increase in lactate content is observed in both uninjected and shRNA-control injected extracts, such an effect was abrogated in shMCT2 injected rats. A ^13^C-incorporation analysis following [1-^13^C]glucose infusion during the stimulation confirmed that the elevated lactate observed during activation originates from newly synthesized [3-^13^C]lactate, with blood-derived [1-^13^C]glucose being the precursor. Moreover, the analysis of the ^13^C-labeling of glutamate in position C3 and C4 indicates that upon activation, there is an increase in TCA cycle velocity for control rats while a decrease is observed for MCT2 knockdown animals. Using *in vivo* localized ^1^H-NMR spectroscopy, an increase in lactate levels is observed in the S1BF area upon whisker stimulation for shRNA-control injected rats but not for MCT2 knockdown animals. Finally, while a robust BOLD fMRI response was evidenced in control rats, it was absent in MCT2 knockdown rats. These data not only demonstrate that glucose-derived lactate is locally produced following neuronal activation but also suggest that its use by neurons via MCT2 is probably essential to maintain synaptic activity within the barrel cortex.

## Introduction

As early as the end of the 19th century, a link between cerebral activity and blood flow modifications has been established [1–3]. Nearly one century later, with the emergence of neuroimaging techniques such as PET or fMRI, an uncoupling phenomenon between glucose and oxygen consumption during brain activation was revealed, suggesting a non-oxidative glucose consumption during neuronal activity [4,5]. A putative explanation for such an uncoupling came in 1994, with the astrocyte-to-neuron lactate shuttle (ANLS) hypothesis [6]. The ANLS proposed a metabolic cooperation between astrocytes and neurons; astrocytes providing glycolytic lactate as an energetic substrate to the more oxidative neurons during brain activation, the release of the neurotransmitter glutamate being the signal for this coupling. Indeed, astrocytes are ideally located between blood vessels, which bring oxygen and glucose to the brain parenchyma, and neurons. Moreover, astrocyte endfeet are in very close proximity with the endothelial cells of brain capillaries. Indeed, brain microvessels are almost entirely covered by astrocyte endfeet in the rat hippocampus [7], suggesting it might constitute a prominent blood-borne glucose uptake site.

The role of astrocytic lactate as an energetic substrate for neurons has been debated during the last twenty years during which numerous *in vitro* experiments were conducted in support of the concept (for a review, see [8,9]). In addition, two *in vivo* experiments have highlighted the importance of glutamate transporters in this astrocyte-neuron metabolic coupling [10,11]. Both studies were conducted in the somatosensory area S1BF, also called the barrel cortex, which can be stimulated by mechanical activation of the whiskers. In the present study, we chose to use also the well-defined whisker-to-barrel pathway [12], which offers unique opportunities for studying metabolism during neuronal activation.

^13^C-nuclear magnetic resonance (NMR) spectroscopy is a powerful technique to investigate neuronal and astrocytic metabolism. Design of ^13^C-NMR studies with ^13^C-enriched substrates is very similar to classical ^14^C-radiolabeling experiments but ^13^C-precursors are rather infused in substrate amounts, and allow to follow the fate of the ^13^C-substrate downstream its metabolism, while ^14^C-precursors are administered in tracer amounts, and, even if frequently used to measure brain activation [13], are trapped after the first glycolysis step. In addition to be non-radiating, ^13^C-NMR presents other important advantages compared to ^14^C, since the detection of ^13^C in the different carbon positions of a specific metabolite does not require further molecule degradation [14]. Moreover, analysis by ^13^C-NMR of homonuclear spin-coupling patterns (shape of the peaks present on the spectrum) allows to determine if two or more ^13^C-atoms are present in the same metabolite molecule. ^13^C-NMR was previously used *in vitro* to demonstrate that lactate was preferentially consumed by neurons compared to glucose when both substrates were present in the medium [15,16], and *ex vivo* experiments have shown that when [3-^13^C]lactate was infused into rats, this ^13^C-labeled substrate entered the brain and was selectively consumed by neurons [17,18]. However, *in vivo* evidence that lactate metabolized by neurons comes from astrocytes has still not been established.

Lactate shuttling requires the expression of specific lactate transporters, called monocarboxylate transporters (MCTs). MCTs are proton-linked membrane carriers involved in the transport of monocarboxylates such as lactate, pyruvate, and ketone bodies. They belong to a large transporter family composed of 14 members based on sequence homologies. MCTs were found in various tissues including the brain in which 3 isoforms, MCT1, MCT2 and MCT4 have been described. Each of these isoforms exhibits a distinct regional and cellular distribution in the rodent brain [19]. At the cellular level, MCT1 is expressed by endothelial cells, ependymocytes, oligodendrocytes and astrocytes. MCT4 expression appears to be specific for astrocytes. In contrast, the predominant neuronal monocarboxylate transporter was found to be MCT2 [20]. Interestingly, part of MCT2 immunoreactivity is located at postsynaptic sites, suggesting a particular link between monocarboxylate utilization and synaptic transmission. In addition to variation in expression during development and upon nutritional modifications, new data indicated that MCT expression in both neurons and astrocytes is regulated at the translational or transcriptional level by various signals, which are also implicated in synaptic plasticity (e.g. BDNF or NO; [21–24]). Moreover, it was shown in a learning and memory task involving the hippocampus that the expression of all three brain MCTs is critical for the acquisition of this paradigm, suggesting that neuron-glia metabolic interactions involving MCTs are critical for synaptic plasticity [25].

The aim of this study was to determine if the neuronal lactate transporter MCT2 is required for proper substrate use by neurons during brain activation. We therefore quantified the brain lactate content by ^1^H-NMR spectroscopy and analyzed the fate of [1-^13^C]glucose by ^13^C-NMR spectroscopy in control, shRNA-control injected rats (called UNIV rats) and MCT2 knockdown rats (called MCT2 rats), at rest or during whisker stimulation. Moreover, we examined the BOLD fMRI response of the somatosensory cortex associated with whisker stimulation.

## Materials and methods

### Animals and infusion techniques

The experimental protocols used in this study were approved by appropriate institutional review committee (Comité pour l'expérimentation animale Bordeaux), meet the guidelines of the responsible governmental agency and were performed by the person having its own animal experiment accreditation (authorization n°5012090-A). No animal utilized for this work became ill or died prior to the experimental endpoint.

*Animals*: Female Wistar rats (200 g, purchased from Janvier Labs, Le Genest Saint Isle, France) were housed in a room with controlled temperature (21–23°C) and a 12/12h normal light/dark cycle. Three groups of animals were studied: control rats (Control rats), rats that previously received a local injection into the somatosensory cortex of a lentiviral vector encoding a shRNA directed against MCT2 (MCT2 rats) or a non targeting sequence (UNIV rats).

### Plasmids and viral vectors

Lentiviral vectors containing the sequence coding for the green fluorescent protein (GFP) as well as either a shRNA directed against MCT2 or a control sequence (shUNIV) have been produced. To generate lentiviral vectors expressing shMCT2 and a control shRNA, oligonucleotides containing 17 nucleotides from the H1 promoter, the sense-strand, a loop, the anti-sense strand for rat and mouse shMCT2 and a universal control sequence (no target in rodent genomes) were synthesized. shMCT2: CTAGTTTCCAAAAATAGGATTAATAGCCAACACTATGACAGGAAGTAGTGTTGGCTATTAATCCTAGGGGATCTGTGGTCTCATACAGAAC, shUNIV: CTAGTTTCCAAAAAGTATCGATCACGAGACTAGTGACAGGAAGCTAGTCTCGTGATCGATACGGGGACTGTGGTCTCATACAGAAC. These oligonucleotides and the primer H1-3F: CACCGAACGCTGACGTCATCAACCCG were used to perform a PCR reaction on the pBC-H1 plasmid [26]. The PCR product was cloned in the pENTR/D-TOPO plasmid (Invitrogen, Saint Aubin, France). The H1-shRNA cassette was then transferred with the LR clonase recombination system (Invitrogen, Saint Aubin, France) in the SIN-cPPT-PGK-GFP-WPRE-LTR-TRE-gateway vector (SIN-CWP-GFP-TRE-gateway) [26].

### Stereotaxic injections

Lentiviral vectors (diluted in phosphate-buffered saline (PBS), 1% BSA to a final concentration of 100 ng p24/μL) were stereotaxically injected bilaterally in the somatosensory cortex. Wistar rats were anaesthetized with a mixture of ketamine (75 mg/kg) and xylazine (10 mg/kg). The temperature was maintained at 37°C during surgery with a heating pad (CMA 450 Temperature Controller from CMA). The cornea was protected with an ophthalmic lubricant. The head was placed in a stereotaxic frame and holes were drilled (at low speed to reduce the heat). Suspensions of lentiviral vectors were injected into the barrel cortex (S1BF area), using a 34-gauge blunt-tip needle linked to a Hamilton syringe by a polyethylene catheter. The stereotaxic coordinates for injection in the S1BF were, from bregma: anteroposterior (AP) 0 mm; lateral (L) 6 mm; and ventral (V) 2 mm from the dura, with tooth bar set at 0 mm. Rats received a total volume of 2 μL per injection site, administered at a rate of 0.2 μL/min. At the end of the injection, the needle was left in place for 5 min before being slowly removed. Both hemispheres received the lentiviral vector injection. The skin was sutured and rats were allowed to recover into a small animal recovery chamber (Harvard Apparatus) before being transferred to their cage.

### Infusion protocol

Experiments were performed in awake animals six weeks after lentiviral vector injection. Animals were slightly held on a Plexiglas support during the stimulation. Before infusion experiments, each animal was accustomed to the experimental set up (at least 3 times, 1h) to avoid any stress. Once rats demonstrated that they lie quietly, they were prepared for the infusion experiment. Right whiskers were mechanically stimulated at a rate of 5 Hz during 1h [13]. To stimulate the maximum of whiskers, they were cut to an equivalent length: 2.5 cm. Infusions were performed in the tail vein during one hour (to reach the isotopic steady-state), during the whisker stimulation. Rats were infused with a solution containing [1-^13^C glucose] (750mM, Cambridge isotope, 99% enrichment) + lactate (sodium salt, 534mM). [1-13C]glucose will labeled different amino acids and metabolites through glycolysis and the TCA cycle [15,27]. Since one mole of [1-13C]glucose gives one mole of labeled [3-13C]pyruvate and one mole of unlabeled pyruvate, there is an isotopic dilution of 50% at the end of glycolysis. From [3-^13^C]pyruvate, [3-^13^C]lactate can be generated by the lactate dehydrogenase (LDH)-catalyzed reaction. Downstream glycolysis, [3-^13^C]pyruvate enters the TCA cycle through two pathways: via pyruvate dehydrogenase (PDH) or via the pyruvate carboxylase (PC) route, present only in astrocytes [28]. Through the PDH pathway, [3-^13^C]pyruvate will be converted to [2-^13^C]acetyl-CoA. It will then enter the TCA cycle to label carbon position 4 of citrate, α-ketoglutarate and glutamate ([4-^13^C]citrate, α-[4-^13^C]ketoglutarate and [4-^13^C]glutamate, from which [4-^13^C]glutamine and [2-^13^C]GABA will be obtained). Then, due to the symmetry of fumarate, the ^13^C will equilibrate between the carbon positions 2 and 3 of this molecule. Therefore, during the next TCA cycle turn, the ^13^C will be found to the same extent in carbon position 2 or 3 of α-ketoglutarate and glutamate (and thereafter glutamine). Through the PC pathway (only in astrocytes), [3-^13^C]pyruvate will follow a different fate and will be converted into [3-^13^C]oxaloacetate. During the next TCA cycle turn, the ^13^C will be located in carbon position 2 of α-ketoglutarate and glutamate (and thereafter glutamine). This enzymatic compartmentation between neurons and astrocytes will thus lead to a different pattern in 13C-labeling, glutamine carbon 2 being more labeled due to the astrocytic PC activity whereas glutamate, present in much higher quantity in neurons, will be equally labeled in carbon positions 2 and 3.

Intravenous infusions were carrying out using a syringe pump that allows a flux such as glucose and lactate concentrations in the blood remain constant (the infusate flow was monitored to obtain a time-decreasing exponential from 15 mL/h to 1.23 mL/h during the first 25 min after which the rate was kept unchanged). At the end of the experiment, a sample of blood was removed; rats were rapidly euthanized by cerebral-focused microwaves (5 KW, 1s, Sacron8000, Sairem), the only way to immediately stop all enzymatic activities and to avoid post-mortem artefacts such as anaerobic lactate production, as already demonstrated [13,29].

Before removing the brain, a hole was drilled at the bregma position, such as a mark was made on the brain, which was then removed and placed in a rat brain matrix that allows precise and reproducible dissection. A 3-mm slice was then cut from bregma and S1BF areas (right-non activated and left-activated) were removed, dipped in liquid nitrogen and kept at -80°C until NMR analyses using a High Resolution at the Magic Angle Spinning (HR-MAS) probe. Both activated and non-activated S1BF brain samples were analyzed in a rotor by HR-MAS NMR spectroscopy on a Bruker Advance 500MHz after perchloric acid extracts (50 μl). Ethylene glycol was added and used as an external reference (1 M, peak at 63 ppm, 2 μl). HR-MAS allows performing spectra with high spectral resolution not only directly on biopsies but also on small perchloric acid extract volumes (50 μL).

### Immunohistochemistry

Three weeks after the lentiviral injection, rats were injected intraperitoneally with a lethal dose of pentobarbital (150g/ml; 1mL/kg, Sigma-Aldrich, Buchs, Switzerland) and then perfused with a solution of 4% paraformaldehyde (Sigma-Aldrich, Buchs, Switzerland) dissolved in 1x PBS at pH 7.4. Brains were dissected, postfixed overnight at 4°C, cryoprotected 24 h in 30% sucrose solution (Sigma-Aldrich, Buchs, Switzerland) and rapidly frozen. Twenty-micrometer thick coronal sections prepared with a microtome-cryostat (Leica MC 3050S) were stored in cryoprotectant (30% ethylene glycol and 35% glycerine in 1x PBS) at -20°C. For immunostaining, sections were washed three times in 1x PBS and blocking of non-specific binding sites was achieved by incubating in 1x PBS containing 5% bovine serum albumin and 0.1% Triton X-100 during 1 h. Immunostainings were carried out overnight at 4°C in PBS containing 5% bovine serum albumin, 0.1% Triton X-100 with either a polyclonal anti-MCT2 (1:500; [30]) or a monoclonal mouse anti-microtubule associated protein (MAP2) antibody (1:200; M4403, Sigma-Aldrich, Buchs, Switzerland). After washing three times with PBS, sections were incubated in a PBS solution with 5% bovine serum albumin and 0.1% Triton X-100 containing a goat Cy3-conjugated anti-rabbit antibody (1:250; #111-165-144, Jackson Immunoresearch, Baltimore, MD, USA) or a donkey Cy5-conjugated anti-mouse antibody (1:400; #715-175-150, Jackson Immunoresearch, Baltimore, MD, USA). After washing twice in PBS, sections were mounted with Vectashield mounting medium (Vector Laboratories, Burlingame, CA, USA). Preparations were then maintained at 4°C until observation with a Zeiss LSM 710 Quasar Confocal Microscope (Zeiss, Feldbach, Switzerland).

Immunofluorescence associated with individual neurons of the somatosensory cortex was quantified by an experimenter blind to the condition of each image using the ImageJ program. The initial quantification was confirmed by a second experimenter blind to the condition of each image. In brief, three sections for each condition were used and selected based on their high number of infected (GFP^+^) neurons. Three images per section were selected to cover the cortical thickness and three neurons on each image were used for quantification for a total of 27 neurons that were distributed randomly in the different cortical layers. The surface of each neuron was manually delineated based on GFP fluorescence or MAP2 immunofluorescence and the intensity of fluorescence as mean of gray value was obtained for each immunofluorescent signal. Background signal was subtracted for each value and then the mean for each immunolabeling was calculated. The value for the MCT2 immunolabeling obtained in the non-infected neurons of the shUNIV condition was considered as 100% and the percentage was calculated for the other conditions. Similarly, the value for the MAP2 immunolabeling obtained in the infected neurons of the shUNIV condition was considered as 100% and the percentage was calculated for the shMCT2 condition.

### Brain perchloric acid extracts

A volume of 1 mL of 0.9 M perchloric acid was added to the frozen S1BF biopsies (around 30 mg) and further sonicated (at 4°C). The mixture was then centrifuged at 5000 g for 15 min (4°C). The brain extract was neutralized with KOH to pH = 7.2, centrifuged again to eliminate potassium perchlorate salts. Supernatant was lyophilized, the final powder was dissolved in 100 μL D2O and bivalent cations were eliminated using Chelex 100 resin beads.

### *Ex vivo* NMR spectroscopy

Experiments were conducted on a Bruker DPX500 spectrometer equipped with a HRMAS probe.

#### ^1^H-NMR spectroscopy

Spectra were acquired at 4°C and the 90° flip angle was measured for each sample. Used parameters were: 8 s relaxation delay, 5000 Hz sweep width and 32 K memory size, water suppression (homonuclear presaturation). The carbon-13 specific enrichment (^13^C-SE) of carbon position 3 for lactate (^13^C-SE lactate C3) was calculated based on the satellite peak areas resulting from the heteronuclear spin-coupling patterns on spectra.

#### ^13^C-NMR spectroscopy

Proton-decoupled 13C-NMR spectra were acquired with the following parameters: 60° flip angle, 20 s relaxation delay, 25063 Hz sweep width and 64 K memory size. Measurements were performed under bi-level broad-band gated proton decoupling and D2O lock at 4°C. Perchloric acid extract spectra were normalized thanks to ethylene glycol and protein contents. After acquisition of each independent sample, perchloric acid extracts of the same group were pooled and lyophilized again. Powder was finally dissolved in 50 μL D2O, pH was adjusted to 6 and another ^13^C-NMR spectrum was acquired (same parameters). This procedure allows a better separation and visualisation of the different carbons between Glu and Gln. Protein content was determined according to the procedure of Lowry et al. [31] using bovine serum albumin as standard.

#### Proton-observed carbon-editing (POCE) sequence

This sequence was used to determine the ^13^C-SE of Glu C4 and Gln C4 using the (13C-1H) heteronuclear multiquanta correlation [32,33]. Briefly, two spectra are acquired: the first scan corresponds to a standard spin-echo experiment without any 13C excitation and a second scan involves a 13C-inversion pulse to get coherence transfer between coupled 13C and 1H nuclei. Subtraction of two alternate scans leads to the editing of 1H spins coupled to 13C spins (scalar coupling constant JCH = 127 Hz). 13C-decoupling was applied during the acquisition to collapse the 1H-13C coupling under a single 1H resonance. Flip angles for rectangular pulses were carefully calibrated on both radiofrequency channels before each experiment. The relaxation delay was 8s for a complete longitudinal relaxation. The 13C-SE was calculated as the ratio of the area of a given resonance on the edited 13C-1H spectrum to its area on the standard spin-echo spectrum. The reproducibility and accuracy of the method were previously assessed using several mixtures of 13C-labeled amino acids and lactate with known fractional enrichments and both were better than 5%.

### *In vivo* MRI and NMR spectroscopy

Experiments were conducted on a 7T Bruker BioSpec system (70/20, Ettlingen, Germany) equipped with a 12-cm horizontal bore, a gradient system capable of 660 mT/m maximum strength and 110 μs rise time. A surface coil (10 mm inner diameter, Bruker) was used for excitation and signal reception.

Animals (4 UNIV and 4 MCT2 rats) were slightly anaesthetized using chloral hydrate (8%, 0.5 ml/100 g body weight). Whisker activation was performed directly into the magnet using an air-pulse system. For this purpose, right whiskers were taped such as a sail was made and this sail was blown at 5 Hz during the acquisition time. In order to place correctly the voxel in the S1BF area, a T_2_-weighted sequence was performed (RARE sequence): 16 slices, 1 mm thick, FOV 5x5 cm. A voxel was then located in the S1BF area (2 x 2.5 x 3 mm) and *in vivo* spectroscopy was performed either at rest or during whisker activation using a PRESS sequence (TE 20 ms, TR 2500 ms, 256 scans, FWHM 14±1 Hz). The lactate peak at 1.32 ppm was quantified using TOPSPIN (Bruker) after phase and baseline corrections.

Finally, functional imaging was performed on 4 control and 4 MCT2 rats. Whisker stimulation was activated (5 Hz, directly into the magnet using an air-pulse system) at half time of the acquisition period. The BOLD response was measured in three slices of 0.7 mm thickness using a single short gradient echo, echo planar imaging sequence (TR = 500ms, TE = 16.096 ms, field of view 25x25 mm², matrix size 96x96 and bandwidth of 33333 Hz). Images were reconstructed and analyzed using FUN TOOL fMRI processing (Bruker software). Quantification was performed by counting the number of activated pixels in the left versus right barrel cortex according to Adamczak et al. [34].

### Statistical analysis

Data are given as mean ± SEM values. They were analyzed with an unpaired *t*-test with Welch's correction for immunostaining quantifications. NMR data (3 groups; control, UNIV and MCT2 rats) were analyzed by an ordinary one-way ANOVA (multiple comparisons) followed by a Bonferroni's correction for post hoc analysis. The level of significance was set at p < 0.05.

## Results

### Downregulation of MCT2 expression in neurons of the rat somatosensory cortex using a lentiviral vector

**Fig 1:**
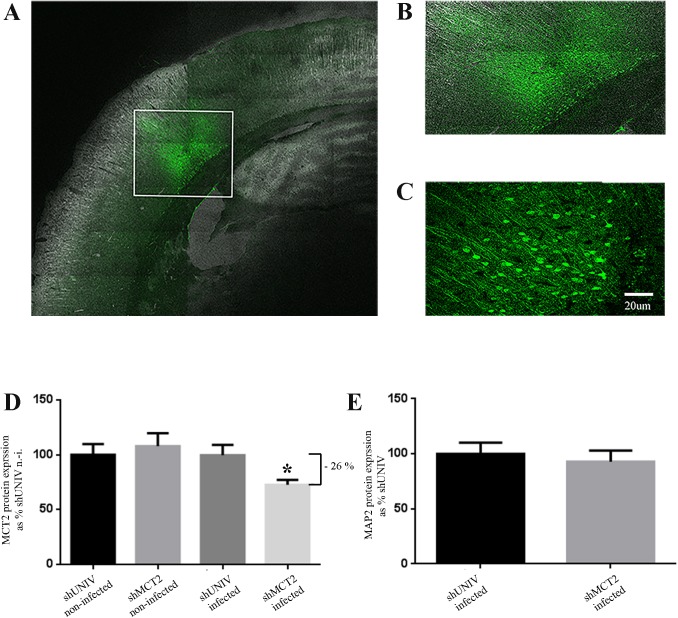
Characterization of the neuronal MCT2 knockdown in the rat somatosensory cortex following the injection of a lentiviral vector to selectively express a shRNA against MCT2. (A, B and C) Fluorescence signal from the expressed Green Fluorescent Protein (GFP) on a coronal section of a rat injected with a lentiviral vector expressing GFP as a marker of infected cells. (A) represents a mosaic of pictures taken at a magnification of 20X with the tilescan function of the confocal microscope. (B) represents a zoom of the area on the mosaic delineated with a white frame (C) represents a picture taken at a magnification of 40X with the confocal microscope. (D) Quantification of the MCT2 immunofluorescence in neurons of the rat somatosensory cortex infected with either a shRNA against MCT2 (shMCT2) or a control shRNA (shUNIV), or non-infected from the same respective sections. The MCT2 immunofluorescence average value for non-infected neurons in sections injected with the shUNIV lentiviral vector was set at 100%. (E) Quantification of the MAP2 immunofluorescence in neurons of the rat somatosensory cortex infected with either a shRNA against MCT2 (shMCT2) or a control shRNA (shUNIV). Data represent mean ± SEM of a total of 27 neurons from three sections for each condition and were statistically analyzed with a Student *t*-test with Welch’s correction. *p < 0.05 vs. shUNIV infected.

As can be seen in Fig 1A, infected cells and fibers are visible as revealed by GFP fluorescence in a portion of the cortex corresponding to the somatosensory cortex. Although superficial layers (layer I-II) appear less labeled, other cortical layers (III-VI) exhibit a strong GFP fluorescence (Fig 1B). At higher magnification, it can be noticed that infected cells appear to be essentially neurons, with numerous cell bodies and processes expressing GFP (Fig 1C).

Prior to perform immunolabeling on sections of infected rats, the specificity of the original MCT2 antibody [30] has been confirmed by pre-adsorption of the primary antibody with the peptide antigen and immunolabeling of cortical sections (S1 Fig) as well as of primary cultures of mouse cortical neurons (S2 Fig) or immunoblot on protein extracts from primary cultures of mouse cortical neurons (S3 Fig). Then, after immunolabeling of sections from injected rats, the MCT2 immunofluorescence was quantified in neurons infected with either the shMCT2 or the shUNIV lentiviral vectors as well as in non-infected neurons in the same sections. No apparent difference in MCT2 immunofluorescence could be observed between non-infected neurons from different animals or between non-infected neurons and neurons infected with the shUNIV vector. In contrast, neurons of the somatosensory cortex infected with the shMCT2 vector exhibited a significant reduction (-26%) of MCT2 expression compared to neurons infected with the shUNIV vector (Fig 1D). Moreover, no significant difference in MAP2 immunofluorescence could be detected between neurons infected with the shMCT2 vector and the shUNIV vector (Fig 1E).

### Comparison of ^1^H-NMR spectra between activated and resting areas

**Fig 2:**
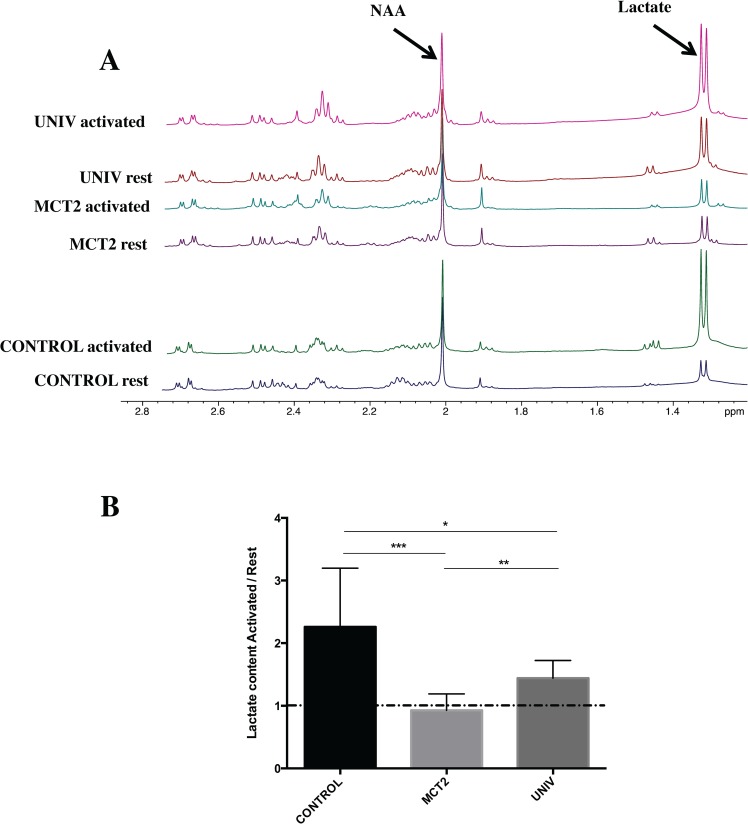
A: *Ex vivo* ^1^H-NMR spectra of the S1BF areas at rest (blue, purple and red) or activated (green, turquoise and pink). Three groups were studied: control rats (control rats, blue and green, n = 12), rats that previously received a local injection into the somatosensory cortex of a lentiviral vector encoding a shRNA directed against MCT2 (MCT2 rats, purple, n = 9 and turquoise, n = 11) or a non targeting sequence (UNIV rats, red and pink, n = 7). B: Ratio of lactate contents between activated and resting states for the three groups. Lactate was quantified from ^1^H-NMR spectra. *** p = 0.0004, * p = 0.045 and ** p = 0.006. NAA, N-acetyl-L-aspartate.

After one hour of right whiskers’ stimulation with concomitant ^13^C-labeled substrate infusion, S1BF areas (right and left) were dissected from microwave-treated brains. HRMAS ^1^H-NMR spectra of S1BF perchloric acid extracts are presented in 2. Protein content and ethylene glycol peak (external reference) were used to normalize spectra. Three groups of rats were studied: control rats (blue, rest S1BF; green, activated S1BF), rats injected with the shMCT2 lentiviral vector, called MCT2 rats (purple, rest S1BF; turquoise, activated S1BF) and rats injected with the shUNIV lentiviral vector, called UNIV rats (red, rest S1BF; pink, activated S1BF). The doublet at 1.32 ppm represents the resonance of the three protons of the methyl group of lactate. By quantifying this doublet, we can directly compare the lactate content in the different conditions and groups. In each individual animal of the group, we were able to measure an increase in lactate content during brain activation but only for the control (n = 14) and UNIV rats (n = 8). In the MCT2 rats (n = 8), no difference was found between resting and activated states. The ratios of lactate contents between activated and resting states were 2.26 ± 0.25, 0.93 ± 0.09 and 1.44 ± 0.11 in control, MCT2 and UNIV rats, respectively (Fig 2B).

### ^13^C-incorporation into brain metabolites in activated and resting areas

**Fig 3:**
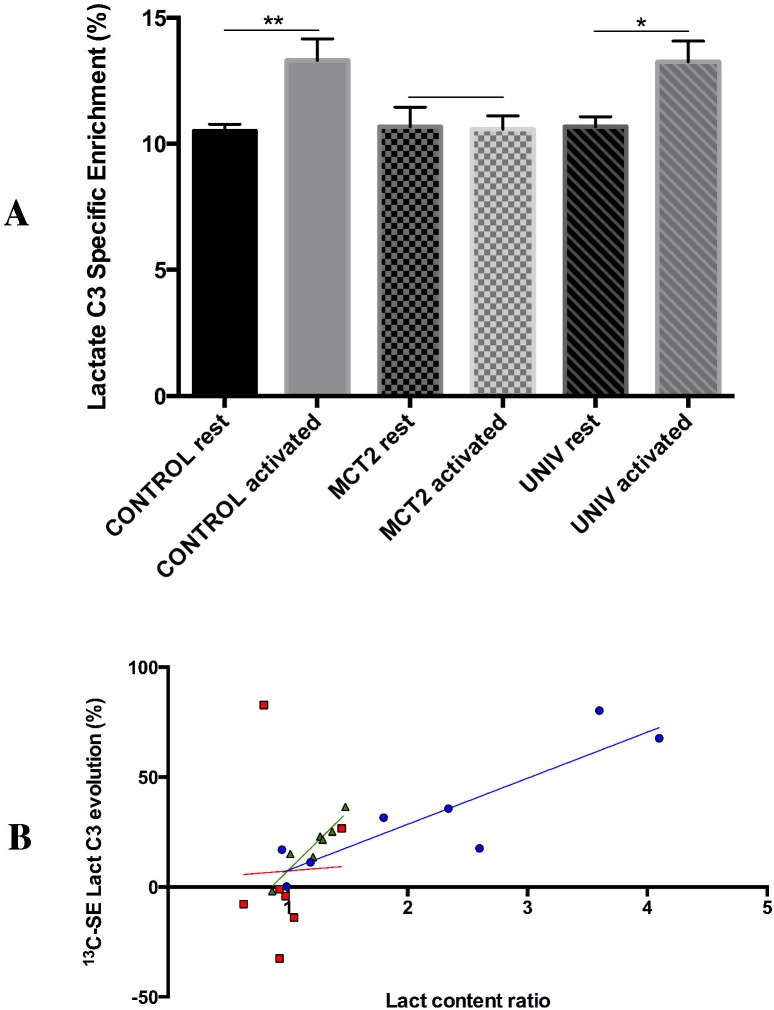
A: ^13^C-Specific enrichments of lactate C3 in the S1BF areas at rest or activated of control, MCT2 and UNIV rats (n = 12 10 and 7, respectively). Values represent the % of carbone-13 that was incorporated into the carbon position 3 of lactate from [1-^13^C]glucose infused in the tail vein during the one-hour right whisker stimulation. ^13^C-Specific enrichments of lactate C3 was quantified from the doublet observed on the ^1^H-NMR spectra. **: p = 0.003, *: p = 0.016. B: Linear regression between increase in lactate content during brain activation (ratio of lactate content between activated and rest states) and evolution in ^13^C-SE Lact C3 (ratio of ^13^C-SE Lact between activated and rest states, %). Each plot represent on individual rat (blue dot, control rats, r^2^ = 0.795; red squares, MCT2 rats, r^2^ = 0.001 and green triangle, UNIV rats, r^2^ = 0.892).

During whisker stimulation, rats were infused with 13C-labeled glucose. The incorporation of ^13^C from glucose into lactate was quantified in the S1BF area of control rats, MCT2 rats and UNIV rats, and compared between resting and activated states. Results are presented in Fig 3A and show a 27% and 24% increase in the lactate C3 specific enrichment (^13^C-SE lactate C3) during brain activation in control and UNIV rats, respectively. No difference in ^13^C-SE lactate C3 between resting and activated states was detected in MCT2 rats. A linear regression was performed between the increase in lactate content and the increase in ^13^C-SE lactate C3 during brain activation using each individual rat value. Fig 3B shows a correlation between these two parameters for control and UNIV rats (r^2^ = 0.80 and 0.89, respectively) but not for MCT2 rats.

^13^C-SE of lactate C3, glutamate C4 and glutamine C4 are presented in Table 1. No statistical difference was found between control and UNIV rats. In MCT2 rats, ^13^C-SE Glu C4 was lower both at rest and during whisker stimulation compared to control and UNIV rats.

**Table 1:** Specific enrichment values (%) of lactate C3, glutamate C4 and glutamine C4 in the S1BF (rest or activated).

%	Control rats		MCT2 rats		UNIV rats	
	rest	activated	rest	activated	rest	activated
**Lac C3**	10.51 ± 0.27	13.31 ± 0.85	10.68 ± 0.77	10.58 ± 0.53*^#^	10.68 ± 0.39	13.00 ± 0.72
**Glu C4**	21.12 ± 0.56	20.40 ± 0.55	**17.04 ± 0.85**^**+**^^**°**^	16.94 ± 0.86*^#^	20.60 ± 0.98	21.63 ± 0.72
**Gln C4**	14.71 ± 0.45	13.14 ± 0.56	14.25 ± 0.97	13.60 ± 0.76	15.53 ± 1.04	16.22 ± 1.52

Three groups were studied: control rats (Lac n = 12, Glu n = 7, Gln n = 7), rats that previously received a local injection into the somatosensory cortex of a lentiviral vector encoding a shRNA directed against MCT2 (MCT2 rats, Lac n = 9–11, Glu n = 9–10, Gln n = 10–11) or a non targeting sequence (UNIV rats, Lac n = 7, Glu n = 7, Gln n = 6).

*: statistically different from the control activated S1BF.

^**#**^: statistically different from UNIV activated S1BF.

^**+**^: statistically different from the control rest S1BF.

**°**: statistically different from UNIV rest S1BF.

**Fig 4:**
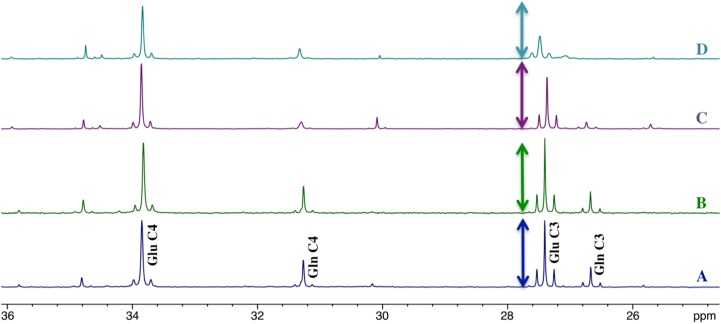
***Ex vivo*** ^**13**^**C-NMR spectra (25–36 ppm) of the pooled S1BF areas at rest (A and C) or activated (B and D) of 8 control rats (A and B) and 8 MCT2 rats (C and D).** Spectra were normalized thanks to the ethylene glycol peak (63ppm) and protein contents. Arrows represent the high of Glu C4 peak.

Since carbon position 4 of glutamate (and thus of glutamine) is labeled during the first TCA cycle turn whereas carbon 2 and 3 are labeled during the second turn, comparison of the incorporation of ^13^C in Glu (Gln) C4 to the one in C3 is an indicator of the TCA cycle turnover rate. Moreover, the largest pool of glutamate being in neurons, the signal corresponding to glutamate peaks will reflect more the neuronal TCA cycle, whereas the glutamine peaks will represent more the astrocytic TCA cycle (glutamine synthetase is present only in astrocytes). Fig 4 represents the part of the 13C-NMR spectra of the S1BF area where Glu and Gln C4 and C3 peaks are located. In control rats, there is a slight increase in ^13^C-incorporation in Glu C3 compared to Glu C4 (height of the peak represented by the line with double arrowheads) during brain activation (Fig 4A and 4B). On the contrary, a decrease is observed in MCT2 rats (Fig 4C and 4D). Incorporation of ^13^C in glutamine is also reduced in MCT2 rats.

Finally, we can compare carbon 2 and 3 of glutamate and glutamine. A ratio lower or close to one is normally observed for glutamate, which was measured in the three groups of rats (Table 2). For glutamine, the C2/C3 ratio value is usually higher than 1, reflecting the pyruvate carboxylase activity present only in astrocytes. Gln C2/C3 ratios were between 1.2 and 1.3 in control and UNIV rats, respectively (no difference between resting and activated states) whereas this ratio was lower than 1 in MCT2 rats.

**Table 2:** Relative enrichment values of glutamate C2/C3 and glutamine C2/C3 C4 in the S1BF (rest or activated).

	Control rats		MCT2 rats		UNIV rats	
	rest	activated	rest	activated	rest	activated
**Glu C2/C3**	0.96 ± 0.04	0.99 ± 0.05	0.83 ± 0.12	0.86 ± 0.08	0.97 ± 0.04	0.97 ± 0.13
**Gln C2/C3**	1.28 ± 0.07*	1.24 ± 0.07*	0.87 ± 0.12^*#*^	0.78 ± 0.13^*#*^	1.20 ± 0.18*	1.25 ± 0.16*

Three groups were studied: control rats (n = 12), rats that previously received a local injection into the somatosensory cortex of a lentiviral vector encoding a shRNA directed against MCT2 (MCT2 rats, n = 8) or a non targeting sequence (UNIV rats, n = 8).

*: statistically different from Glu C2/C3.

^**#**^: statistically different from control and UNIV rats.

### *In vivo* experiments in the barrel cortex: ^1^H-NMR spectroscopy and BOLD fMRI

**Fig 5:**
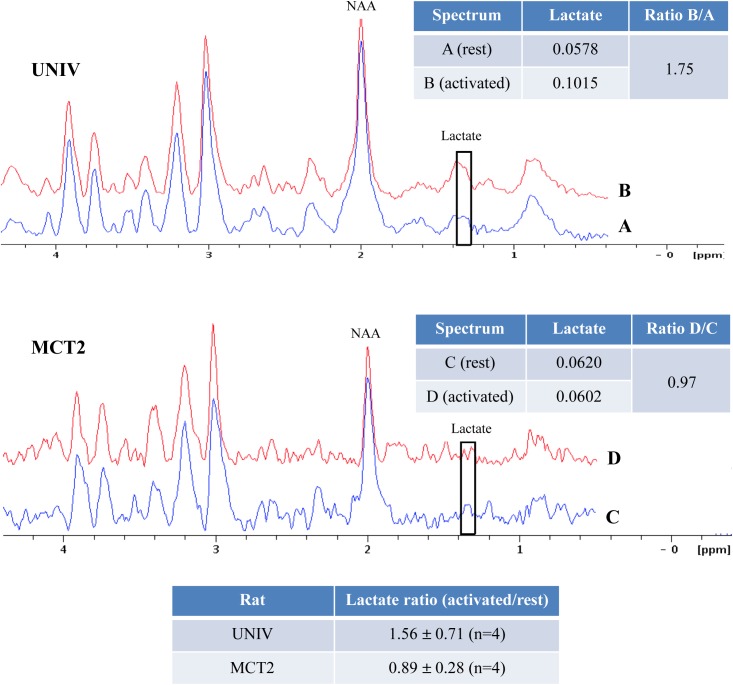
***In vivo*** ^**1**^**H-NMR spectra of the S1BF areas at rest (A and C) or activated (B and D) of UNIV rats (A and B, n = 4) and MCT2 rats (C and D, n = 4).** Spectra shown represent one typical experiment and were normalized thanks to the NAA peak (2.01 ppm).

*In vivo* localized ^1^H-NMR spectroscopy was performed in the S1BF area (voxel size 2x2.5x3 mm) of UNIV and MCT2 rats (Fig 5). An increase in lactate was observed during brain activation in UNIV rats but not in MCT2 rats, confirming the *ex vivo* results. Ratios of lactate (activated/rest) were 1.56 ± 0.40 (n = 4) and 0.89 ± 0.16 (n = 4), respectively.

**Fig 6:**
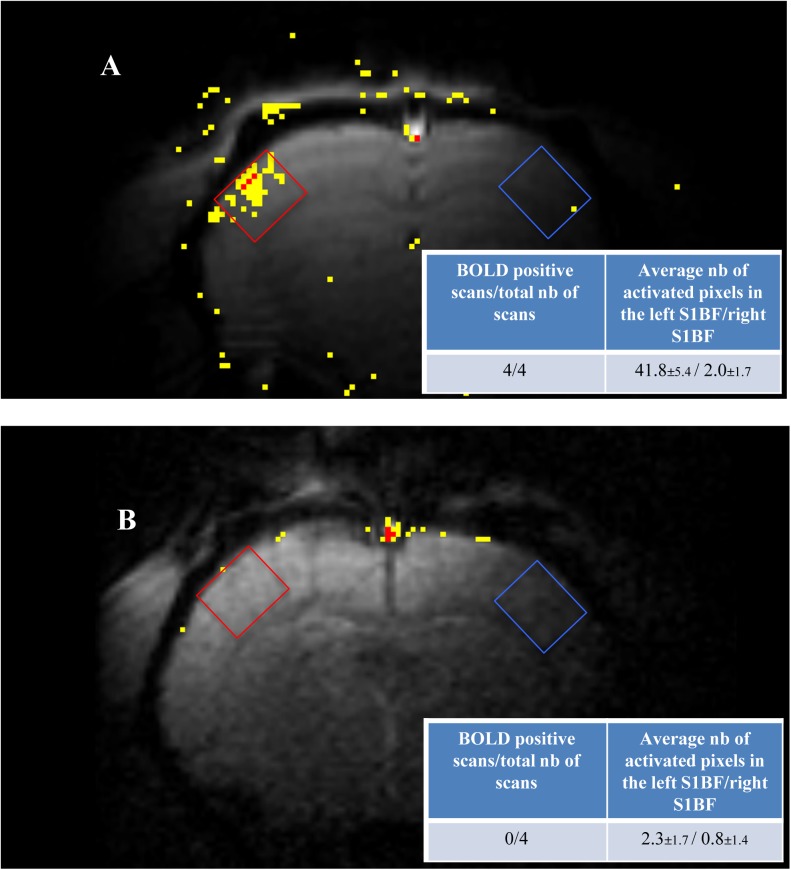
**BOLD-fMRI of the S1BF areas of control rats (A, n = 4) and MCT2 rats (B, n = 4) during right whisker activation.** Images shown represent one typical experiment. Quantifications of the 4 experiments are presented in the tables. The red square represents the location of the voxel (present on 3 slices) in which the in *vivo* spectroscopy was performed and the number of activated pixel was counted in both red (activated S1BF) and blue (rest S1BF) voxels.

Finally, BOLD fMRI was performed on stimulated animals (Fig 6). In control rats, the right whisker stimulation led to a BOLD signal in the left barrel cortex. Surprisingly, this signal was no more visible in MCT2 rats.

## Discussion

### Lactate accumulation in the barrel cortex following whisker stimulation arises from metabolism of blood-borne glucose

In control rats, we observed an increase in lactate content in the left S1BF area (barrel cortex) following right whisker stimulation. The lactate content was measured *ex vivo* by high resolution NMR spectroscopy after brain microwave fixation. This technique was shown to be the most suitable to avoid any post mortem metabolism, and thus suppress any anaerobic lactate production during the time needed to remove the brain from the skull. Indeed, it stops reactions within the brain in less than 1s and allows to get an instant "picture" of the metabolite content at the precise time the focused microwaves are applied [13]. Such an increase in cortical lactate confirms previous observations made using NMR spectroscopy both in humans during a visual task [35–37], and *ex vivo* in rats [13]. Moreover, it is consistent with the idea that lactate production is positively correlated with cerebral activity [38] as it was shown using different anaesthetics (pentobarbital, α-chloralose and morphine) with distinct sedative potencies and thus different brain electrocortical activities [39]. As such, an increase in lactate content can be due either to an increase in lactate synthesis or to a decrease in lactate consumption during whisker activation. To distinguish between these two hypotheses, the use of ^13^C-NMR spectroscopy is of particular interest. Compared to microdialysis or ^1^H-NMR spectroscopy studies, which allow only lactate quantification, ^13^C-NMR spectroscopy allows to follow a ^13^C-labeled precursor and thus track its fate. While [1-^13^C]glucose was infused in control rats during whisker stimulation, we observed an increase in the specific enrichment of [3-^13^C]lactate (^13^C-SE lactate C3). This means that the increase in total lactate, measured by ^1^H-NMR spectroscopy, is linked to the increase in ^13^C-incorporation into lactate. Therefore, this lactate represents newly synthesized lactate, blood-borne [1-^13^C]glucose being the precursor. This conclusion is supported by the correlation observed between variation in lactate content (^1^H-NMR data) and variation in ^13^C-SE lactate C3 in control rats (Fig 3B). Higher the lactate content increases, higher is the ^13^C-SE lactate C3. This correlation was further confirmed in UNIV rats (with a smaller inter-individual variability). However, even if we measured an increase in lactate content between rest and activated states in UNIV rats, this increase was less compared to control rats. This was mainly due to a higher lactate content in the barrel cortex of the UNIV rats at rest, since the lactate level in the activated barrel cortex of the UNIV rats was similar to the one measured in control rats (see Fig 2A). A possible explanation may lie in the experimental conditions. Compared to control and MCT2 rats, UNIV rats were less quiet on the Plexiglas support during the [1-13C]glucose infusion. This state might have contributed to elevate resting lactate levels. Nevertheless, increases in ^13^C-SE lactate C3 between rest and activated states were similar in control and UNIV rats, indicating a similar 13C-lactate production from 13C-glucose.

### Blunted lactate response in MCT2 rats

MCT2 belongs to a small group of membrane carriers involved in the transport of lactate, pyruvate and ketone bodies. MCT2 is the main monocarboxylate transporter isoform present on neurons [20,30,40]. In MCT2 rats, no rise in lactate content was observed during whisker stimulation. This result can have two distinct explanations. First, lactate could be produced by neurons. The downregulation of MCT2 could suppress lactate export and as a consequence its neuronal synthesis, the LDH enzyme working at equilibrium. Lactate accumulation within the neuron could lead to an inhibition of glycolysis via some feedback mechanisms or simply by intracellular acidification and thus cause a reduction in neuronal lactate production *per se*. Nevertheless, considering the recent *in vivo* observation of a lactate gradient from astrocytes to neurons using two-photon microscopy and a FRET lactate sensor [41], it seems unlikely that neurons are the source of lactate. Therefore, we should consider a second possibility, i.e. that lactate is coming from astrocytes. Indeed, it has been demonstrated that astrocytic glycolysis is activated by the uptake of the neurotransmitter glutamate [6,42,43]. However, if one considers that lactate is coming from the astrocytic compartment and is produced as a consequence of whisker stimulation, with the downregulation of MCT2, neurons would rather be prevented to use it. In such case, one could have predicted a lactate accumulation in the tissue, which was not the case. An alternative explanation could be that the signal that stimulates astrocytic glycolysis, i.e. glutamate, is lacking, meaning that synaptic activity is strongly reduced in the S1BF of MCT2 rats. In accordance with this possibility it was recently shown in rat brain slices that the inhibition of MCTs by 4-CIN significantly decreased the amplitude of EPSCs [44]. The authors concluded that astrocyte-neuron lactate shuttling to presynaptic and postsynaptic elements is necessary for the integrity of excitatory synaptic transmission. In addition, the essential role of MCT2 in long-term memory formation, and thus most likely on synaptic activity, was also demonstrated using an antisense oligodeoxynucleotide to knockdown the neuronal lactate transporter [25].

### Reduction of neuronal TCA cycle rate in MCT2 rats is coherent with reduced neuronal activity

When [1-^13^C]glucose is infused in rats, the carbon 13 will be incorporated during the first TCA cycle turn on carbon position 4 of glutamate. Carbon position 3 of glutamate will be labeled only during the next TCA cycle turn [15,45]. Therefore, the ratio of ^13^C-incorporation into Glu C3 relative to the one into Glu C4 reflects the neuronal TCA cycle rate, since the large glutamate pool is located in neurons [46,47]. In parallel, the ratio of ^13^C-incorporation into Gln C3 relative to the one into Gln C4 reflects the astrocytic TCA cycle rate, since glutamine is synthesized only in astrocytes [28]. Fig 4 is a higher magnification view of the ^13^C-NMR spectra area where the Glu C4, Gln C4, Glu C3 and Gln C3 peaks are located. To better visualize the changes, the height of the Glu C4 peak has been represented by a line with two arrowheads next to the Glu C3 peak on the spectra. While in control rats the Glu C3 peak height increased relative to the Glu C4 peak with whisker stimulation, this effect was not observed in MCT2 rats (rather the contrary, it decreased with whisker activation). Moreover, even at rest, the height of the Glu C3 peak is lower relative to the Glu C4 peak, indicating a slower basal neuronal TCA cycle rate in MCT2 rats compared to control rats. A link between neuronal activity and TCA cycle rate was already established in previous studies, using pentobarbital, α-chloralose or morphine to reach different brain electrocortical activities. Sibson et al. found a correlation between V_TCA_ and neuronal activation; the more the anaesthetic lowered brain electrocortical activity, the more V_TCA_ decreased [48]. Using the same protocol, a decrease in the ratio Glu C3/C4 was measured from morphine to pentobarbital [49]. Thus, the observed decrease in the Glu C3 peak height relative to the Glu C4 peak in MCT2 rats therefore strongly supports the idea that synaptic activity is reduced in the barrel cortex of these animals.

### Reduced glutamate recycling by astrocytes in MCT2 rats is consistent with reduced synaptic activity

In Fig 4, we can also clearly observe a reduction of ^13^C-incorporation into glutamine in the MCT2 rats (Gln C4 and C3 peaks in spectra C and D, compared to the corresponding ones in spectra A and B). The knockdown of the neuronal lactate transporter has therefore not only an impact on neuronal metabolism but also on the astrocytic one. Since [4-^13^C]Gln is coming from the consumption of [1-^13^C]glucose by astrocytes, the decrease in ^13^C incorporation into Gln C4 indicates a reduction in glucose metabolism in MCT2 rat astrocytes. Estimation of the PC activity corroborates this result. Indeed, quantification of Gln C2/C3 ratio is a suitable approach to evaluate this specific astrocytic anaplerotic enzyme activity; ratio higher than one indicating ^13^C-incorporation through the PC activity (this pathway leads to direct ^13^C-incorporation into glutamine carbon position 2 [27]). On the contrary, since no PC activity is present in neurons, the Glu C2/C3 ratio is always lower than 1 [50]. Table 2 shows Glu C2/C3 and Gln C2/C3 values. In control (and UNIV) rats, Gln C2/C3 ratios were higher than 1, demonstrating, as expected, the astrocytic PC activity, which was not detected in the neuronal compartment (Glu C2/C3 < 1). However, in MCT2 rats, Gln C2/C3 ratio was smaller than 1, indicating that the PC activity was no more detected in the barrel cortex of these animals. This result strengthens our hypothesis that neuronal activity is strongly reduced in the S1BF area of the MCT2 rats. Indeed, if the neurotransmitter glutamate is no more released into the synaptic cleft, the signal for astrocytes to increase their glycolysis is suppressed, and, as a consequence, the glutamate-glutamine cycle will be drastically reduced, since replenishment of neuronal glutamate by astrocytic glutamine is no more needed. This will directly impact on the activity of the anaplerotic astrocytic PC, as observed in our study. A strong decrease in both glutamate-glutamine cycle and glutamine synthesis rates was already observed when brain activity was reduced [39].

### Neuronal lactate uptake via MCT2 and its utilization might be necessary to maintain synaptic transmission: Preliminary *in vivo* evidence

*In vivo* NMR spectroscopy was also conducted in UNIV and MCT2 rats. Compared to *ex vivo* ^1^H-NMR, *in vivo* localized ^1^H-NMR spectral resolution was much lower and the lactate peak was hindered by the lipid signal, which makes difficult the quantification of the lactate signal at 1.32 ppm. However, despite these difficulties, these *in vivo* results confirmed the *ex vivo* ones obtained with the same paradigm. An increase in lactate content was obtained with brain activation, which was no more observed in MCT2 rats. This increase was less (ratio = 1.56) compared to the one measured on *ex vivo* spectra (2.26), which could be mainly due to the fact that for *in vivo* NMR animals were slightly anaesthetized. However, such results not only support the idea that lactate is produced locally upon activation, but it also suggest that neuronal lactate uptake via MCT2 might be necessary to sustain synaptic activity. Lactate is not only taken up by neurons to be used as an additional energy substrate, it can also act via lactate-sensitive G-protein coupled receptors. However, when lactate binds to this receptor, it reduces neuronal excitability [51]. In our case, we observed a decrease in neuronal activity when lactate levels were reduced following MCT2 knockdown. Thus, it seems unlikely that this is the mechanism that explains our findings. However, independently of its effect on the receptor, the fact that lactate levels are reduced (no matter which cell type produces it) might contribute to the reduction in synaptic activity. Indeed, it was shown that lactate (via its metabolism) potentiates NMDA-mediated currents [52]. To further gain evidence that MCT2 is essential for cerebral activation, we performed BOLD fMRI both on control and MCT2 rats. We observed that the BOLD response in the left S1BF was absent in MCT2 rats compared to control rats. Therefore, the absence of lactate increase during brain activation in MCT2 rats is most likely linked to a reduction of synaptic activity within the barrel cortex.

### Technical considerations and perspectives

In order to progress in our understanding of metabolic interactions between neurons and astrocytes, further technical developments will be required, e.g. to improve spectral resolution, time resolution and peak quantification, with the use of adabatic pulses to produce a more uniform excitation profile and of LCModel to quantify the peaks with a better accuracy for example. Moreover, in this study we chose a lentiviral approach, which led to a 26% reduction in MCT2 expression at the protein level. This reduction was sufficient to suppress the brain lactate increase in the barrel cortex during whisker stimulation, to modify ^13^C-SE lactate C3 and glutamate C4 as well as the ratios of different carbons in glutamate and glutamine. A 40% decrease in protein content was described previously to be sufficient to observe a clear phenotype in the case of other target proteins, suggesting that it is not necessary to achieve prominent suppression of protein expression to be able to obtain significant alterations in functional and/or metabolic responses [53,54]. However, it would be desirable to improve the efficiency of the viral vector approach in order to increase the reliability and extent of the effects observed. In this regard, the development of other viral vectors, derived from the AAV family, has led to a better efficiency to deliver and express for example an shRNA with distinct cell type specificities [55,56]. Using such novel tools to either better reduced neuronal MCT2 expression, but also target the astrocytic lactate transporters (MCT1 and 4), should constitute the next step in order to shed light on the role of lactate transporters in the metabolic cooperation between neurons and astrocytes during brain activation.

Notwithstanding, our present data confirm that lactate is synthesized most likely by astrocytes in the brain from blood-borne glucose during cerebral activity. Moreover, and quite unexpectedly, they also strongly support the idea that neuronal lactate use through its transport via MCT2 is necessary to maintain synaptic transmission.

## Supporting information

S1 Fig Specificity of MCT2 immunolabeling in the rodent cortex. Strong MCT2 immunofluorescence (in red) associated with the neuropil as well as numerous neuronal cell bodies in the mouse cortex (left panel). Important reduction of the immunofluorescence signal in the mouse cortex when the primary antibody had been incubated with the peptide antigen prior to immunolabeling (right panel).
(TIF)

S2 Fig Specificity of MCT2 immunolabeling in primary cultures of rodent cortical neurons. Strong MCT2 immunofluorescence visible in both cell bodies and neuronal processes in the entire population of mouse cortical neurons in culture (left upper and lower panels, at 20x and 40x magnification, respectively). After incubation of the primary antibody with the peptide antigen, important reduction of the immunofluorescence signal upon immunolabeling of a similar preparation (right upper and lower panels, at 20x and 40x magnification, respectively).
(TIF)

S3 Fig Specificity of MCT2 antibody by immunoblotting on protein extracts from primary cultures of mouse cortical neurons. Western blot showing the unique band at 40 kDa recognized by the MCT2 antibody (left panel) in three distinct protein extracts from primary cultures of mouse cortical neurons. After adsorption of the primary antibody with the peptide antigen, immunoblot showing the absence of the 40 kDa signal (right panel).
(TIF)

S1 File Materials and Methods and references of supporting figures. (DOCX)

S1 Data Raw data. (DOCX)

## Abbreviations

AAV: Adenoviral-associated virus
ANLS: Astrocyte-neuron lactate shuttle
BOLD: Blood oxygen level-dependent
BDNF: Brain-derived neurotrophic factor
4-CIN: alpha-cyano-4-hydroxycinnamate
CTL: Control
EG: Ethylene glycol
EPSC: Excitatory postsynaptic current
fMRI: Functional magnetic resonance imaging
FRET: Förster resonance energy transfer
FWHM: Full width at half maximum
GABA: Gamma amino butyric acid
GFP: Green fluorescent protein
Gln: Glutamine
Glu: Glutamate
HRMAS: High-resolution magic angle spinning
Hz: Hertz
KD: Knockdown
KW: Kilowatt
LACT: Lactate
LDH: Lactate dehydrogenase
MAP2: Microtubule associated protein 2
MCT: Monocarboxylate transporter
NAA: N-acetyl-L-aspartate
NMR: Nuclear magnetic resonance
NO: Nitric oxide
PBS: Phosphate-buffered saline
PC: Pyruvate carboxylase
PCR: Polymerase chain reaction
PDH: Pyruvate dehydrogenase
PET: Positron emission tomography
PGK: Phosphoglycerate kinase
POCE: Proton-observed 13C-editing
S1BF: Somatosensory cortex barrel field
SE: Specific enrichment
SEM: Standard error of the mean
shRNA: Short hairpin ribonucleic acid
7T: 7 Teslas
TCA: Tricarboxylic acid
TE: Time of excitation
TR: Time of relaxation
UNIV: Universal (non-targeting sequence)
V_TCA_: Tricarboxylic acid cycle rate
WPRE: Woodchuck hepatitis posttranscriptional response element
