# Supplementary figures and images for: A neuronal MCT2 knockdown in the rat somatosensory cortex reduces both the NMR lactate signal and the BOLD response during whisker stimulation
Source: PLoS One. 2017 Apr 7;12(4):e0174990. doi: 10.1371/journal.pone.0174990 (PMC5384673; doi:10.1371/journal.pone.0174990)

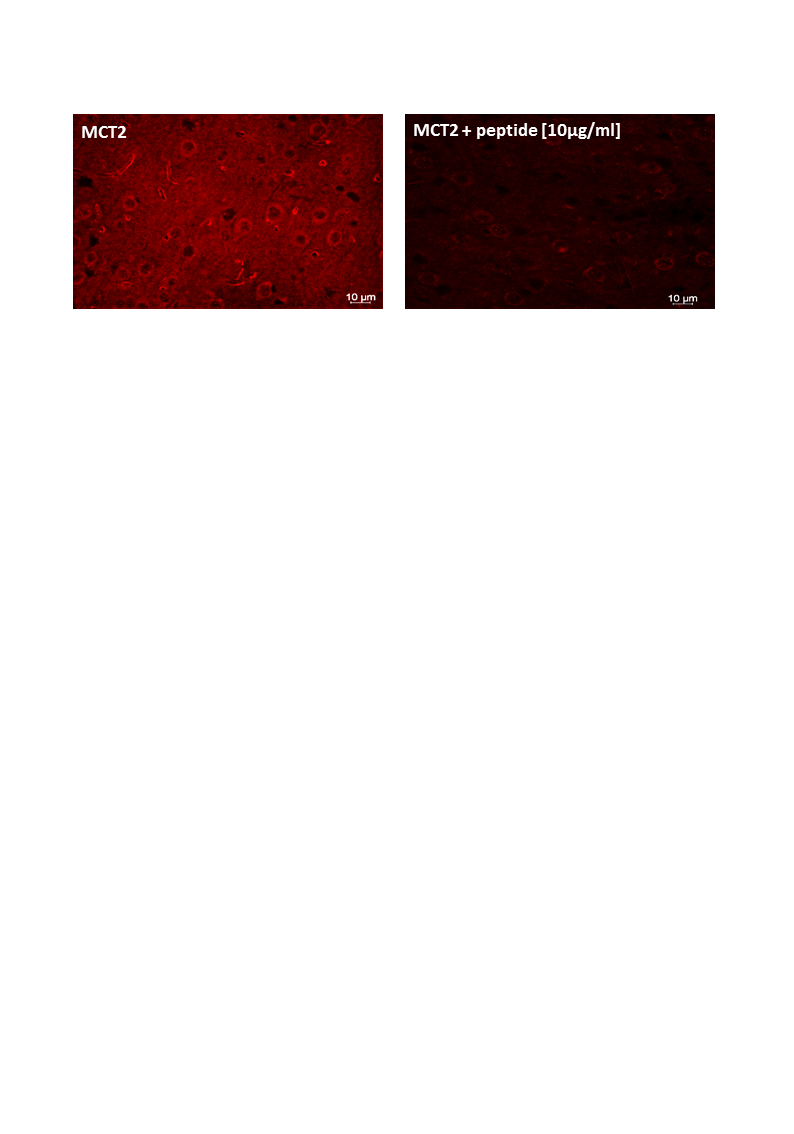

Supplement: S1 Fig — Strong MCT2 immunofluorescence (in red) associated with the neuropil as well as numerous neuronal cell bodies in the mouse cortex (left panel). Important reduction of the immunofluorescence signal in the mouse cortex when the primary antibody had been incubated with the peptide antigen prior to immunolabeling (right panel). (TIF) [file pone.0174990.s001.tif]

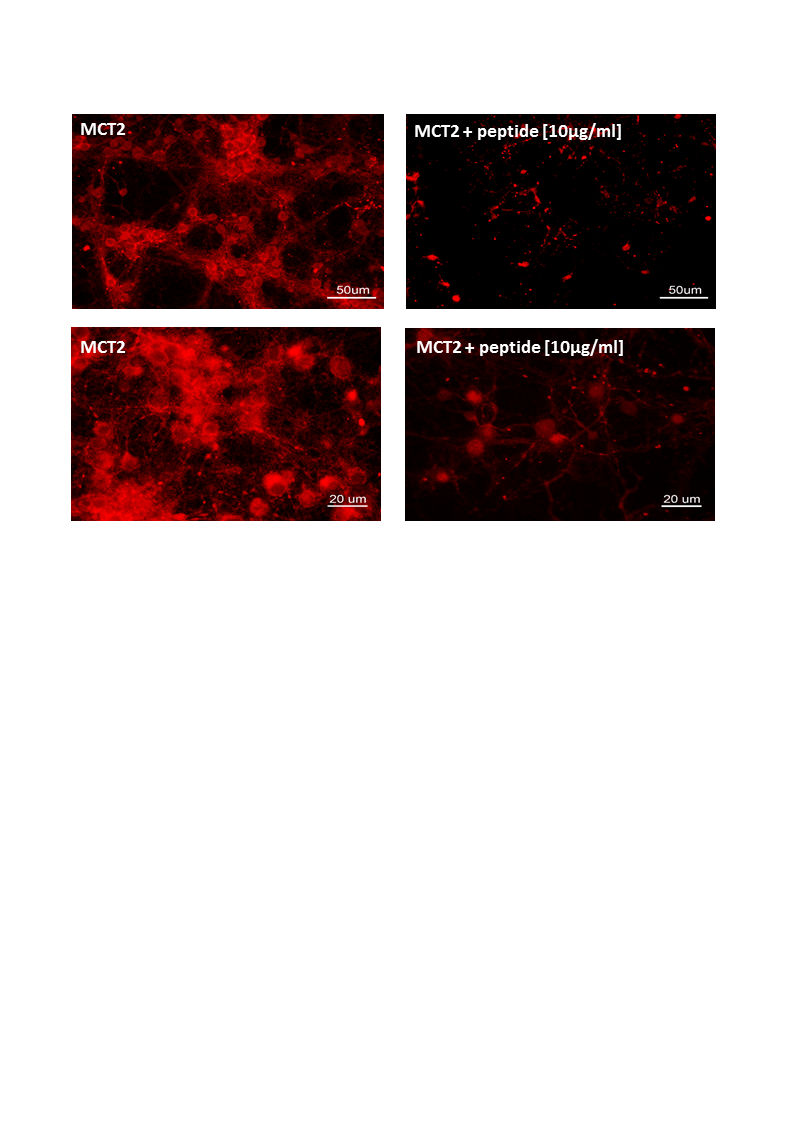

Supplement: S2 Fig — Strong MCT2 immunofluorescence visible in both cell bodies and neuronal processes in the entire population of mouse cortical neurons in culture (left upper and lower panels, at 20x and 40x magnification, respectively). After incubation of the primary antibody with the peptide antigen, important reduction of the immunofluorescence signal upon immunolabeling of a similar preparation (right upper and lower panels, at 20x and 40x magnification, respectively). (TIF) [file pone.0174990.s002.tif]

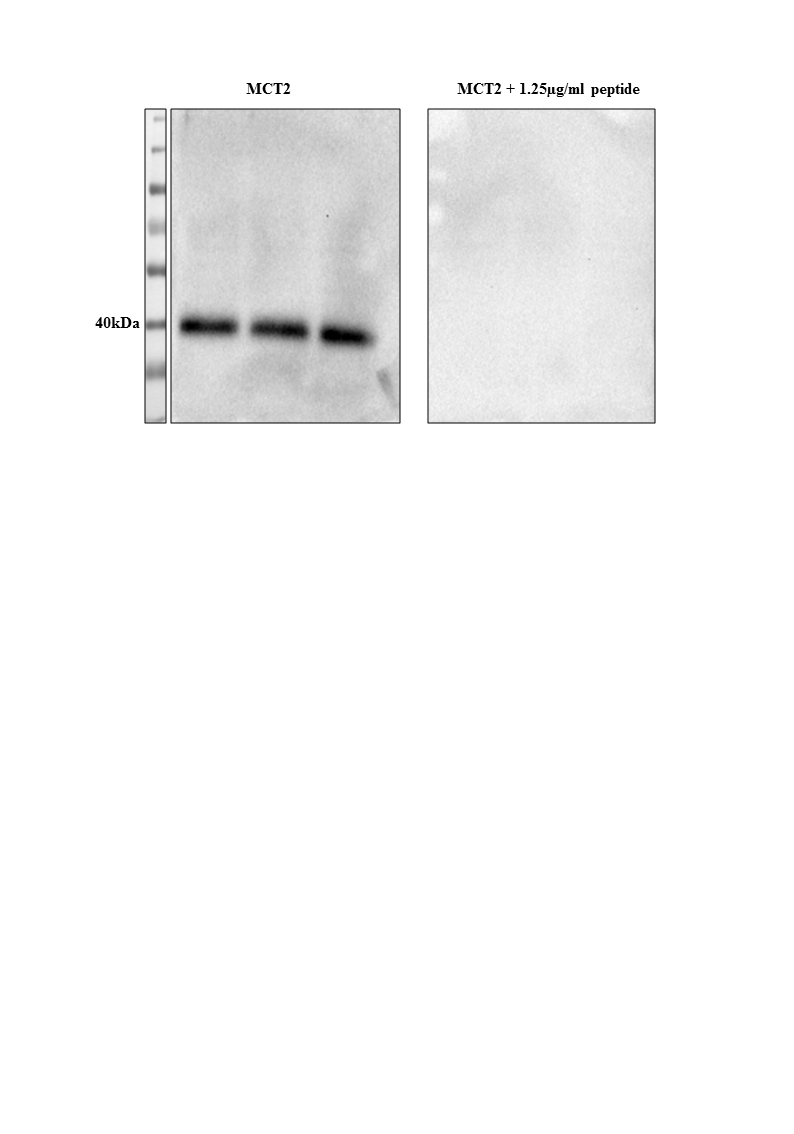

Supplement: S3 Fig — Western blot showing the unique band at 40 kDa recognized by the MCT2 antibody (left panel) in three distinct protein extracts from primary cultures of mouse cortical neurons. After adsorption of the primary antibody with the peptide antigen, immunoblot showing the absence of the 40 kDa signal (right panel). (TIF) [file pone.0174990.s003.tif]
