# Supplementary material for: A neuronal MCT2 knockdown in the rat somatosensory cortex reduces both the NMR lactate signal and the BOLD response during whisker stimulation
Source: PLoS One. 2017 Apr 7;12(4):e0174990. doi: 10.1371/journal.pone.0174990 (PMC5384673; doi:10.1371/journal.pone.0174990)
Supplement: S1 File — (DOCX) [file pone.0174990.s004.docx]

**Supporting information**

**Materials and Methods**

Experiments were conducted in accordance with the Swiss Federal Guidelines for Animal Experimentation and were approved by the Cantonal Veterinary Office for Animal Experimentation (Vaud, Switzerland).

**Preparation of mouse brain sections for immunostaining**

For immunofluorescence labeling, the brain from an adult mouse C57Bl6 was collected after performing perfusion with 4% PFA. The brain was post-fixed in the same solution overnight at 4°C and cryoprotected by successive immersions in 15 and 30% sucrose in PBS. Twenty µm free-floating coronal brain sections were prepared with a microtome.

**Primary cultures of mouse cortical neurons**

Primary cultures of cortical neurons were prepared from embryonic day 17 (E17) OF1 mice embryos (Janvier Labs) as described previously (Yang J et al. 2014). After decapitation and brain dissection, cortices were dissociated by enzymatic reaction with a solution containing Papain 200U (BioConcept, # LS003126), Penicillin-Streptomycin (Gibco, # 15140), L-cysteine 1mM (Sigma, # C7880), DNase I 1000U (BioConcept, # LS002138) in Hank's Balanced Salt Solution (HBSS homemade). Neurons were plated on poly L-ornithine (15mg/L) precoated dishes (Westen blot) or glass coverslips (Immunostaining) at an average density of 4 * 10^5^ cells/cm^2^ for immunoblot (Western Blot) and 4.5 * 10^5^ cells/cm^2^ for immunofluorescence labeling. Cells were maintained in Neurobasal medium which contains 25mM glucose (Gibco, #12348-017) supplemented with B27 (Gibco, # 17504), GlutaMAX (Gibco, #350505), Penicillin (50 U/mL) and Streptomycin (50µg/mL) at 37°C in a humidified atmosphere containing 5% CO_2_ and 95% air. Cultures were used at day 7 *in vitro.* These culture conditions typically produced 93% pure neuronal cultures, as assessed by microtubule-associated protein 2 (neuronal marker) and GFAP (astrocytic marker) co-immunostaining (Bélanger M et al. 2011).

**Specificity of MCT2 immunostaining**

For immunostaining of brain sections, free-floating coronal mouse brain sections were treated with PBS buffer containing 5% BSA, 0.1% Triton X-100 during 1 h to block non-specific sites. For immunostaining of cultured neurons, after removal of the culture medium, cells were carefully rinsed in PBS at 37°C and directly post-fixed in an ice-cold paraformaldehyde fixative (4% in PBS for 30 min at room temperature). Fixed cells were treated with PBS buffer containing 5% BSA, 0.1% Triton X-100 for 1 h at room temperature to block nonspecific sites.

The MCT2 blocking peptide CNTHNPPSDRDKESSI was used to test the antibody specificity in immunofluorescence staining and immunoblot. Pre-absorption of the polyclonal (rabbit) antibody raised against MCT2 (1:500 dilution; Pierre et al. 2000) with the MCT2 blocking peptide [10µg/ml] for 1 h at room temperature was performed prior to dilute it in PBS buffer containing 5% BSA, 0.1% Triton X-100. Brain sections or cultured neurons grown on coverslips were incubated overnight at 4°C with either the non-adsorbed or the adsorbed MCT2 antibody. After washing three times with PBS, sections or cultured neurons were incubated in a PBS solution containing a donkey Cy3-conjugated anti-rabbit antibody (1:500 dilution; # 715-165-152, Jackson Immunoresearch) for 2 h at room temperature. After rinsing three times in PBS, sections and coverslips were mounted with Vectashield mounting medium (Reactolab SA,# H-1000). Preparations of coronal sections were then maintained at 4°C until observation with a Zeiss LSM 710 Quasar Confocal Microscope while coverslips were examined and photographed with an Axioplan2 microscope (Nikon microscope Eclipse 90i) using epifluorescence with an appropriate filter.

**Western blot**

Neurons in each culture dish were washed twice in ice-cold PBS and homogenized in 80 μL of extracting buffer containing the following: Tris-HCl, pH 6.8, 80mM; EDTA (ethylene diamine tetraacetic acid), 5mM; SDS, 5% and a mixture of protease inhibitors (Complete 11257000, Roche,Basel,Switzerland). About 15µg of proteins were denatured at 95°C during 5min in SDS-PAGE sample buffer (60 mM Tris–HCl pH 6.8, 5% SDS, 6.6% glycerol, 5 mM EDTA, 5% β-mercaptoethanol and 0.1% bromo-phenol blue). Samples were loaded onto polyacrylamide gels composed of a 10% acrylamide running gel and a 4.5% acrylamide stacking gel using an Electrophoresis Unit (Bio-Rad, Cressier, Switzerland). After electrophoresis, proteins were transferred onto nitrocellulose membranes (0.45mm; #162-0115, Bio-Rad, Cressier, Switzerland) using a Transblot semi-dry transfer cell (Bio-Rad). For protein detection, membranes were incubated in a blocking solution of Tris-buffered saline supplemented with Tween-20 (TBST; Tris-HCl, pH 7.5, 50 mmol/L; NaCl, 150 mmol/L; and Tween-20, 0.1%) containing 10% skim milk powder and 1% BSA for 1 h at room temperature. Pre-absorption of the polyclonal (rabbit) antibody raised against MCT2 (1:1000 dilution) and the MCT2 blocking peptide [1.25µg/ml] for 1 h at room temperature was performed prior to dilute it in TBS-T 0.1% containing 1% skim milk powder. Blots were then incubated overnight at 4°C with either the adsorbed or the non-adsorbed MCT2 antibody. Membranes were washed three times in TBS-T 0.1% and they were subsequently incubated 2 h at room temperature with horseradish peroxidase-conjugated donkey anti-rabbit IgG (1:10,000 dilution; GE Healthcare, #NA9340V, Glattbrugg, Switzerland). After being washed three times in TBS-T 0.1%, blots were processed using Immun-Star^TM^-WesternC^TM^ Chemiluminescent Kit (#170–5070, Bio-Rad, Cressier, Switzerland). Chemiluminescence detection was performed with the ChemiDoc^TM^ XRS System (#170–8070, Bio-Rad, Cressier, Switzerland) and quantification was made with the ImageLab 3.0 software (Bio-Rad, Crissier, Switzerland).

**References**

Bélanger M, Yang J, Petit JM, Laroche T, Magistretti PJ, Allaman I (2011) Role of the glyoxalase system in astrocyte-mediated neuroprotection. J Neurosci 31:18338-18352.

Pierre K, Pellerin L, Debernardi R, Riederer BM, Magistretti PJ (2000) Cell-specific localization of monocarboxylate transporters, MCT1 and MCT2, in the adult mouse brain revealed by double immunohistochemical labeling and confocal microscopy. Neuroscience 100:617-627.

Yang J, Ruchti E, Petit JM, Jourdain P, Grenningloh G, Allaman I, Magistretti PJ (2014) Lactate promotes plasticity gene expression by potentiating NMDA signaling in neurons. Proc Natl Acad Si USA 111:12228-12233.
