# Supplementary material for: A neuronal MCT2 knockdown in the rat somatosensory cortex reduces both the NMR lactate signal and the BOLD response during whisker stimulation
Source: PLoS One. 2017 Apr 7;12(4):e0174990. doi: 10.1371/journal.pone.0174990 (PMC5384673; doi:10.1371/journal.pone.0174990)
Supplement: S1 Data — (DOCX) [file pone.0174990.s005.docx]

**^13^C specific enrichments**


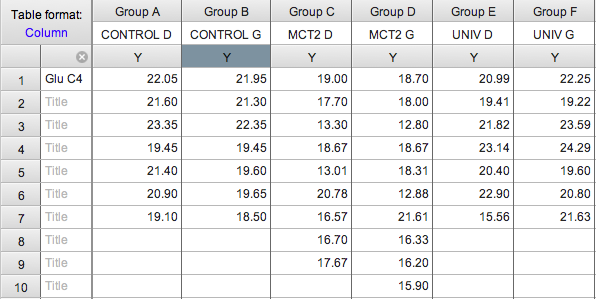

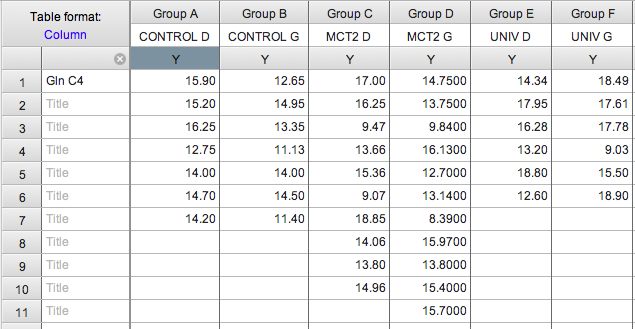

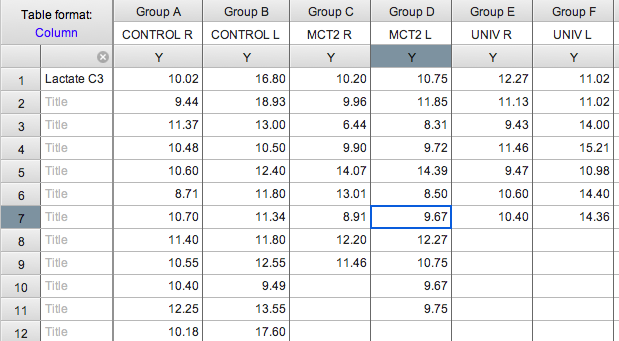


**^13^C relative enrichments**


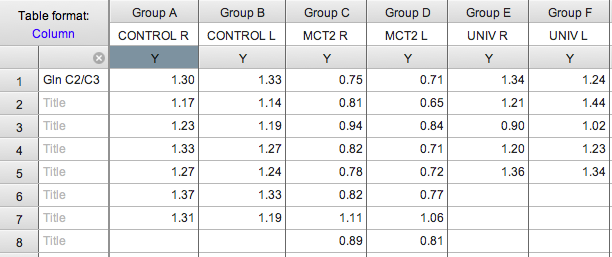

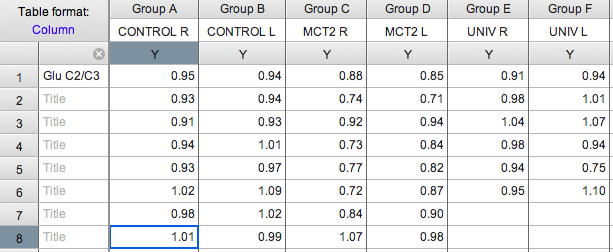


**ANOVA for lactate**


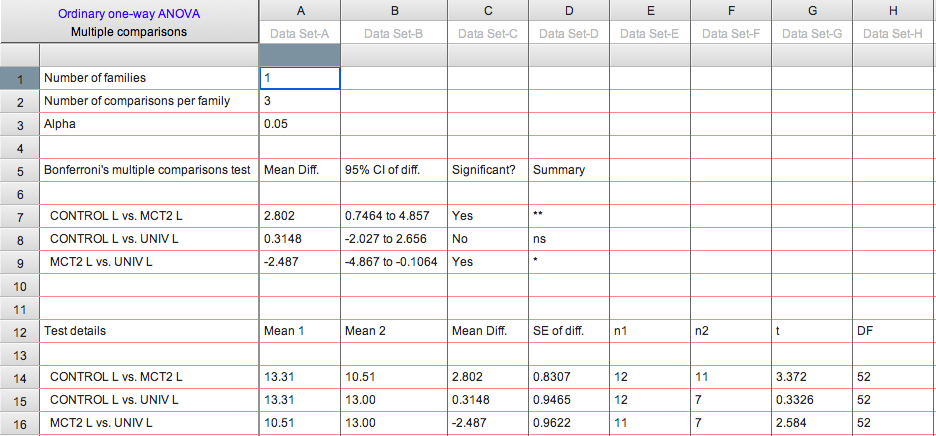

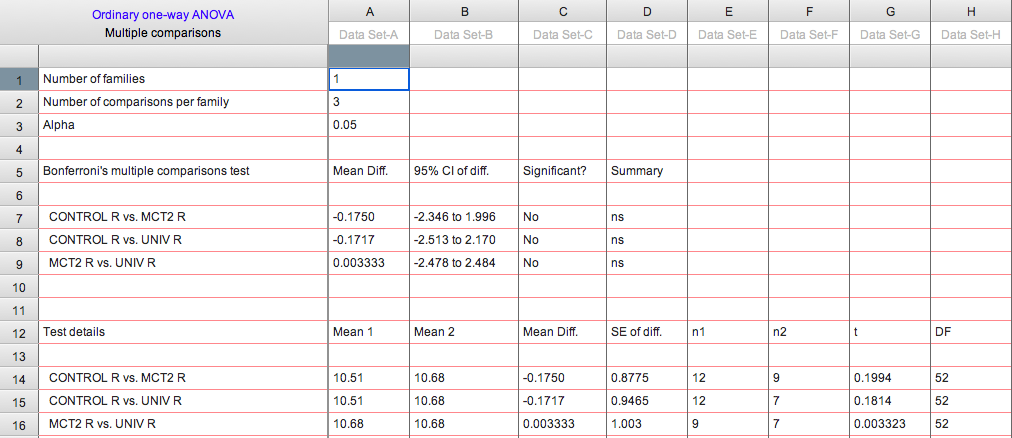


**Typical ^13^C-NMR spectrum of one barrel cortex (UNIV)**


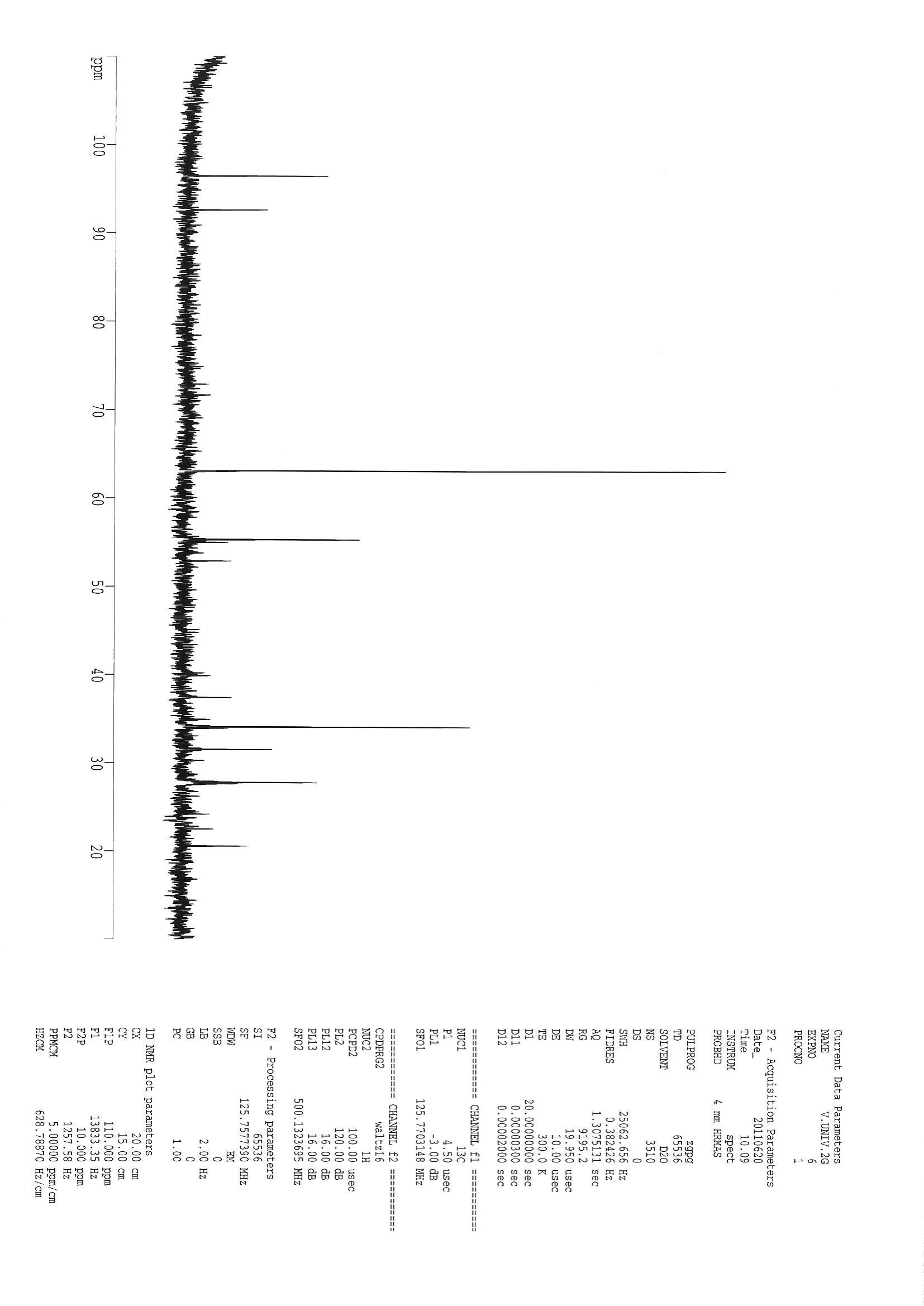


**Immunohistochemistry quantifications**

| **ShUNIV - Infected cells - Raw Data** | | |
| --- | --- | --- |
| Slide | Mean gray value - BKG ( 1st investigator) | Mean gray value - BKG (2nd investigator) |
| Slide 1_1 | 20,03033333 | 7,955666667 |
|  | 19,75433333 | 12,91066667 |
|  | 16,50733333 | 11,06566667 |
| Slide 1_2 | 10,96566667 | 11,96533333 |
|  | 13,51366667 | 17,30133333 |
|  | 11,53166667 | 9,822333333 |
| Slide 1_3 | 16,112 | 11,92733333 |
|  | 18,356 | 9,890333333 |
|  | 10,671 | 9,439333333 |
| Slide 2_1 | 9,032666667 | 20,33133333 |
|  | 14,64666667 | 16,02333333 |
|  | 12,80366667 | 19,86333333 |
| Slide 2_2 | 18,228 | 16,83133333 |
|  | 14,902 | 16,33833333 |
|  | 14,017 | 15,29433333 |
| Slide 2_3 | 12,302 | 18,76266667 |
|  | 11,178 | 20,73266667 |
|  | 7,985 | 21,72966667 |
| Slide 3_1 | 5,601333333 | 5,739333333 |
|  | 5,894333333 | 5,066333333 |
|  | 4,939333333 | 6,102333333 |
| Slide 3_2 | 4,64 | 5,125 |
|  | 4,451 | 4,924 |
|  | 4,46 | 4,114 |
| Slide 3_3 | 3,672 | 4,918 |
|  | 4,377 | 4,584 |
|  | 4,929 | 3,404 |
| Average | **10,94448148** | **11,56155556** |

| **ShMCT2 - Infected cells - Raw Data** | | |
| --- | --- | --- |
| Slide | Mean gray value - BKG ( 1st investigator) | Mean gray value - BKG (2nd investigator) |
| Slide 1_1 | 4,052666667 | 5,932333333 |
|  | 2,918666667 | 6,875333333 |
|  | 5,658666667 | 5,024333333 |
| Slide 1_2 | 9,369333333 | 3,42 |
|  | 8,449333333 | 4,309 |
|  | 9,217333333 | 2,475 |
| Slide 1_3 | 10,62566667 | 9,628333333 |
|  | 9,488666667 | 9,396333333 |
|  | 13,81066667 | 7,399333333 |
| Slide 2_1 | 8,636666667 | 12,19733333 |
|  | 7,816666667 | 13,20133333 |
|  | 7,591666667 | 13,43333333 |
| Slide 2_2 | 5,368 | 8,099666667 |
|  | 6,676 | 11,84866667 |
|  | 5,829 | 5,218333333 |
| Slide 2_3 | 7,814333333 | 9,231333333 |
|  | 7,143333333 | 8,180333333 |
|  | 8,735333333 | 7,612333333 |
| Slide 3_1 | 12,16533333 | 6,344666667 |
|  | 11,62133333 | 8,321666667 |
|  | 10,85733333 | 4,120666667 |
| Slide 3_2 | 8,086 | 8,282666667 |
|  | 11,076 | 6,430666667 |
|  | 7,758 | 10,14566667 |
| Slide 3_3 | 7,137333333 | 7,606 |
|  | 3,787333333 | 8,689 |
|  | 6,419333333 | 9,38 |
| Average | **8,078148148** | **7,881617284** |

| **ShUNIV - Infected cells - in %** | | |
| --- | --- | --- |
| Slide | Mean gray value - BKG ( 1st investigator) | Mean gray value - BKG (2nd investigator) |
| Slide 1_1 | 183,0176548 | 68,8113864 |
|  | 180,4958359 | 111,6689411 |
|  | 150,827916 | 95,71088089 |
| Slide 1_2 | 100,1935696 | 103,4924174 |
|  | 123,4747091 | 149,6453764 |
|  | 105,3651257 | 84,95684933 |
| Slide 1_3 | 147,2157455 | 103,1637419 |
|  | 167,7192294 | 85,54500548 |
|  | 97,50119289 | 81,64414631 |
| Slide 2_1 | 82,5317004 | 175,8529225 |
|  | 133,8269583 | 138,5915006 |
|  | 116,9874214 | 171,8050243 |
| Slide 2_2 | 166,5496902 | 145,5801795 |
|  | 136,1599453 | 141,3160474 |
|  | 128,0736783 | 132,2861207 |
| Slide 2_3 | 112,4036805 | 162,2849674 |
|  | 102,1336645 | 179,3241971 |
|  | 72,95914396 | 187,9476041 |
| Slide 3_1 | 51,17952224 | 49,64153228 |
|  | 53,85667054 | 43,82051627 |
|  | 45,13081174 | 52,7812482 |
| Slide 3_2 | 42,39579561 | 44,32794511 |
|  | 40,66889791 | 42,58942472 |
|  | 40,75113113 | 35,58344706 |
| Slide 3_3 | 33,5511555 | 42,53752859 |
|  | 39,99275806 | 39,64864397 |
|  | 45,03639582 | 29,44240491 |
| Average | **100** | **100** |








**BOLD and in vivo spectroscopy quantifications**

|  | nb pixel in right S1BF | nb pixel in left S1BF |
| --- | --- | --- |
| control 1 | 46 | 0 |
| control 2 | 47 | 5 |
| control 3 | 34 | 2 |
| control 4 | 40 | 1 |
|  | 41,75 | 2 |
|  | 5,359473699 | 1,732050808 |
|  | nb pixel in right S1BF | nb pixel in left S1BF |
| MCT2 1 | 1 | 0 |
| MCT2 2 | 5 | 0 |
| MCT2 3 | 1 | 3 |
| MCT2 4 | 2 | 0 |
|  | 2,25 | 0,75 |
|  | 1,712393549 | 1,419727086 |

|  | lactate ratio (activated/rest) |
| --- | --- |
| UNIV 1 | 1,75 |
| UNIV 2 | 1,09 |
| UNIV 3 | 2,04 |
| UNIV 4 | 1,37 |
|  | 1,5625 |
|  | 0,399803988 |
|  | lactate ratio (activated/rest) |
| MCT2 1 | 0,97 |
| MCT2 2 | 0,69 |
| MCT2 3 | 0,84 |
| MCT2 4 | 1,07 |
|  | 0,8925 |
|  | 0,156809 |
|  |  |

**Voxel localization:**

Voxel size: 2 x 2.5 x 3 mm, located on 3 slices, corresponding to approximately coordinates -0.36, -2.40 and -4.36 on the Paxinos atlas.
